# Should Surgeons Mask While Operating? The Deadliness of a Sneeze

**Published:** 1914-06-13

**Authors:** 


### SHOULD SURGEONS MASK WHILE OPERATING?

### The Deadliness of a Sneeze.

During the last few years the fashion has become almost general among surgeons of donning a sterilised gauze or muslin mask before operating, shaped so as to cover the whole head except the eyes and the nose. The object of this is to prevent pathogenic organisms which may be exhaled by the surgeon (or his assistant) from entering the wound and there giving rise to sepsis or other mischief of zymotic origin. Some surgeons even go so far as to request that the anæsthetist, as well as the assistant, shall wear a mask; others wear a mask only for certain operations, as in joint and bone operations; others deride it as a fad.

At the May meeting of the Devon and Exeter Medico-Chirurgical Society Mr. A. L. Candler read a paper* embodying the results of his experiments as to the necessity or otherwise of these masks. He used *bacillus prodigiosus* in his researches, and gives three sensible reasons for his choice of this organism. First, it is not found in the air or in the human mouth in ordinary circumstances; thus chance contamination of the culture media during the experiments can be excluded. Secondly, its colonies develop a red colour, and are there on readily identified by the naked eye. Thirdly, it is not pathogenic, so that the experiments were free from risk to those who carried them out.

Mr. Candler procured a culture of this microbe, then, and mixed it with water until a turbid, slightly pink, suspension was obtained. He filled his mouth with a quantity of this and gargled it. After expelling the fluid he proceeded to breathe, speak, cough, or sneeze over a series of sterile culture plates, with and without a mask. In each case the experimenter's mouth was held about eighteen inches away from the plate, and each experiment was conducted in a different room in order to avoid any air infection of the plate from a previous experiment. The mask used was of the popular butter-muslin type, but reinforced with plain gauze over the nostrils and mouth. In some of the tests six layers of gauze were used, and in others eight. In other words, the conditions were closely approximated to those which obtain during a surgical operation, except that an exposed culture medium took the place of the operation wound.

The results showed, broadly speaking, that during quiet breathing and speaking no bacilli are conveyed to the culture plates from the mouth. Prolonged coughing expelled only a few bacilli, which four layers of gauze were insufficient to trap, but eight layers intercepted them all. Sneezing, however, is a far more effective way of distributing oral bacteria. A single sneeze gave rise to 130 colonies on a circular plate of three and a-half inches diameter; that is, 130 bacilli settled on the plate. How many more were discharged into the vicinity but missed the plate can only be speculated; but the number must have been very large. Even eight layers of gauze failed to filter off all the organisms during prolonged sneezing, though they did catch most of them. It is, of course, to be recognised that no modern surgeon would either cough or sneeze with his face a foot and a-half from an operation wound. If the impulse to do either became overpowering he would turn round away from the patient before letting himself go. Mr. Candler deduced from his experiments that a mask such as he described (and showed to the meeting) is both necessary and efficient, and is a safeguard to the patient.

In the discussion subsequent to the paper a diversity of opinion was made manifest as to the need for the mask when operating. Obviously, it was open to those who decry the mask to make much of the experiments, which showed that during quiet breathing and talking there is no risk of microbes reaching the wound from the surgeon's mouth. One surgeon said he wore a mask only in unusually heavy operations, where profuse perspiration might occur. This left him open to the natural retort from the reader of the paper that if a mask is necessary in some cases it is advisable in all. We may say, from the practical standpoint, that a light mask is an advantage, provided it does not incommode the operator; but that no one could accuse a surgeon of *malapraxis* in refusing to wear one, and that the fashion is one which is easily pushed to a ridiculous excess.

* *Lancet*, May 30, 1914, p. 1542.

